# Factors associated with preeclampsia and the hypertensive disorders of pregnancy amongst Indigenous women of Canada, Australia, New Zealand, and the United States: A systematic review and meta-analysis

**DOI:** 10.1007/s11906-025-01327-6

**Published:** 2025-02-20

**Authors:** Jacqueline Stephens, Eleonora Dal Grande, Tayla Roberts, Marianne Kerr, Celine Northcott, Tahlia Johnson, Jessie Sleep, Courtney Ryder

**Affiliations:** 1 College of Medicine and Public Health, Flinders Health and Medical Research Institute, Flinders University, Adelaide, Australia; 2 Centre for Kidney Research, University of Sydney, New South Wales, Australia; 3 College of Nursing and Health Sciences, Flinders University, Adelaide, Australia; 4 Far West Community Partnerships, Ceduna, Australia

**Keywords:** Pregnancy, Indigenous, Hypertension, Pregnant women [MESH], Pre-eclampsia [MESH], Risk factors [MESH]

## Abstract

**Purpose of the Review:**

Preeclampsia and the Hypertensive Disorders of Pregnancy (HDP) occur more frequently amongst Indigenous women and can have short- and long-term impacts on maternal and infant health and wellbeing. To understand factors associated with increased risk for Indigenous women a systematic review and meta-analysis was conducted. The PRISMA guidelines were adhered to, and the review protocol was registered on PROSPERO (Registration CRD42023381847). EndNote, Covidence and Excel were used to screen and extract data, with studies assessed using JBI critical appraisal tools.

**Recent Findings:**

Seven studies from Canada, Australia, and the United States (none from New Zealand) were included in this review. Meta-analysis showed women classified as overweight (OR 1.32, 95% CI: 1.09–1.60), obese (OR 1.88, 95% CI: 1.57–2.25), or having high mean BMI (MD 3.02 95% CI: 1.72–4.31), high mean systolic blood pressure (MD 15.19 95% CI: 12.83–17.541), or high mean diastolic blood pressure (MD 15.26 95% CI: 13.05–17.47), pre-pregnancy diabetes (OR 3.63, 95% CI: 1.66–17.94), or high microalbuminuria (OR 2.76, 95% CI: 1.40–5.43) were more likely to be diagnosed with preeclampsia. Smoking (OR 0.77, 95% CI: 0.58–1.03), alcohol consumption (OR 1.70, 95% CI: 0.76–3.81), and gestational diabetes (OR 1.74, 95% CI: 0.90–3.37) were not associated with preeclampsia.

**Summary:**

Understanding factors associated with increased preeclampsia/HDP risk amongst Indigenous women is important to minimising adverse perinatal events and future health complications. This review demonstrates current gaps in the evidence, specifically in relation to social, economic, and environmental factors.

**Supplementary Information:**

The online version contains supplementary material available at 10.1007/s11906-025-01327-6.

## Introduction

Preeclampsia, one of the main Hypertensive Disorders of Pregnancy (HDP), is a leading cause of maternal and perinatal morbidity and mortality [1], and is attributed to 70,000 maternal and 500,000 foetal deaths annually [2, 3]. It causes severe complications in pregnancy, including preterm labour and emergency caesarean section, foetal growth restriction, and lower birthweight in babies [4, 5]. Preeclampsia occurs in 4.6% pregnancies world-wide with regional estimates varying between 1% and 5.6% [6]. The effects of preeclampsia can extend beyond pregnancy, with women who experience this condition facing an increased risk of developing chronic health issues later in life, including end stage renal disease, liver failure, cardiovascular diseases (CVD), ischaemic heart disease, and type 2 diabetes [3, 7]. Children who were exposed to preeclampsia in utero have an increased risk of developing cardiovascular disease, diabetes mellitus, and insulin-resistance syndromes in adulthood [3, 8].

Preeclampsia is defined as a new onset of high blood pressure in pregnancy typically associated with proteinuria and is usually detected after 20 weeks of gestation [1, 2, 9]. There is no cure; only delivery or termination of pregnancy resolves preeclampsia. Therefore, early detection is essential to reduce the associated complications by providing effective and appropriate management to avoid adverse pregnancy outcomes [2, 10, 11]. Prognostic preeclampsia factors include preeclampsia or hypertension in previous pregnancies, having pre-existing chronic kidney disease, hypertension, diabetes, or an autoimmune disorder [10, 12]. Other potential factors include first pregnancy, maternal age > 40 years, an interval of 10 years or more between pregnancies, body mass index (BMI) ≥ 35 kg/m^2^), polycystic ovary syndrome (PCOS), family history of preeclampsia, assisted reproductive technologies use, and a multi-foetal pregnancy [10, 12]. However, some factors commonly associated with other cardiovascular and metabolic conditions, such as alcohol consumption and smoking, have not been confirmed as associated with preeclampsia or HDP because of inconsistent results across studies [13, 14]. Although not as thoroughly researched as health factors, socioeconomic factors, such as education level [15], and environmental factors, such as air pollution [16, 17], have also been linked to increased risk of preeclampsia and HDP [18].

Research suggests Indigenous women globally have higher rates of preeclampsia/HDP when compared to other population groups. For example, in Australia between 2016–18 about 6.6% of pregnant Aboriginal and Torres Strait Islander women had HDP compared to 5.5% non-Indigenous pregnant women [19]. However, amongst another cohort of Aboriginal and Torres Strait Islander women, over a third (36%) had experienced HDP [20]. While the incidence of preeclampsia among Aboriginal and Torres Strait Islander women has decreased over time [21], this gap persists internationally for Indigenous women. For example, the proportion of preeclampsia amongst pregnant American Indian and Alaskan Native women in the United States is estimated to be between 7–11% [22], while preeclampsia affects only 3–5% of pregnancies in the white American population.

Ongoing colonisation and land dispossession within countries such as Canada, Australia, New Zealand, and the United States can be directly linked to disease burden, poverty, and disadvantage faced by Indigenous peoples through all stages of life [23–25]. Colonisation is an ongoing structure of domination of one culture over another using a range of policies and processes [26], resulting in abrupt lifestyle changes and the loss of traditional practices [24]. The increased rate of Indigenous women with conditions such as preeclampsia can be attributed to ongoing trauma caused by colonisation, such as forced migration, systematic racism and discrimination [22]. These experiences impact on interactions and trust with health services, and perpetuate ongoing barriers to healthcare, including access to adequate antenatal care. For example, Indigenous women have been shown to have a lower number of antenatal visits and attendance rates in the first trimester of pregnancy. [27, 28] These low rates of healthcare attendance have been shown to be associated with social and socio-economic influences, including residential remoteness, lack of access to appropriate services and treatment, language barriers, and racism [29–31]. Furthermore, culturally appropriate antenatal care typically does not align with Western biomedical models, with Indigenous women less likely to engage early in their pregnancy with antenatal care that is not linked to their spiritual beliefs or the traditional ways of giving birth [32–35].

To date, preeclampsia and HDP research often includes a diversity of women. However, research including a subgroup of Indigenous women, or which only includes Indigenous women, often have sample sizes too small for reliability. Compiling such studies into a systematic review will lessen this issue, which has been done for other pregnancy outcomes. For example, a systematic review explored factors associated with poor birth outcomes for Indigenous women, found several health and social factors (such as pre-existing medical conditions, decreased access to prenatal care, high rates of multiple births, young maternal age, and low educational achievement) increased the risk of poor birth outcomes, such as low birth weight [36]. However, considerable variation between the methods and results of the individual studies included in the analyses impacts on the certainty of the findings [36]. Furthermore, the majority of previous studies are limited by the focus of their knowledge systems. Their focus on a Western Biomedical approach to knowledge generation, with no Indigenous knowledges integrated into their research methodologies, results in findings that lack relationality.

This is the first systematic review to investigate the association between health and socioeconomic factors and the development of preeclampsia amongst Indigenous women of Canada, Australia, New Zealand, and the United States. Although we acknowledge Indigenous groups of these countries are culturally distinctive, experiencing different social environments, political climates and levels of reconciliation, they were selected with significant similarities in processes of colonisation, which continues to affect their health and wellbeing [26]. Identifying these factors will assist in prevention of short and long term consequences of preeclampsia for Indigenous women [11].

## Methods

We conducted a systematic review and meta-analysis of prognosis studies, following PRISMA guidelines [37]. The systematic review protocol was registered on PROSPERO (Registration CRD42023381847) [38].

### Eligibility Criteria

Inclusion criteria were based on PEO (population, exposure, outcome) components [37]. The population was Indigenous women from Canada, Australia, New Zealand and the United States and their islands, including Hawaii. This includes the First Nations, Metis, and Inuit people of Canada, Aboriginal and Torres Strait Islanders of Australia, Māori of New Zealand, and Native Americans of the United States [26]. The primary outcome of the study was a diagnosis of preeclampsia. We determined papers reporting HDP could also be included if a lack of studies entirely on preeclampsia were found. The diagnosis was to be made by a medical professional based on criteria or guidelines set by international, or the country’s, standards at the time of the study, such as the International Society for the Study of Hypertension in Pregnancy [11]. The exposure was any measurable health or socioeconomic factors linked to the outcome (preeclampsia or HDP), such as sociodemographic indicators (age, parity, income, or education level) and clinical indicators (diabetes, obesity, or BMI). The study needed to have risk factor data on Indigenous women diagnosed with and without preeclampsia or HDP. Other criteria for inclusion were published (no date restriction) and accessible quantitative studies that were either randomized controlled trials (RCT), cohort, cross sectional, or case control studies conducted on humans.

### Search Strategy

The original search was performed on 24th January 2023 with translation into five databases: Ovid Medline, Ovid Emcare, APA PsycINFO, Scopus and Cochrane Library (see Supplementary Materials). Initial keywords were gathered from related papers, systematic reviews, and Medical Subject Headings (MeSH). Keywords included in the search strategy were related to *Indigenous people* and *preeclampsia*. A variety of spellings and syntax were used. No other limits were applied. The results from each database were initially passed through a two-step process using EndNote [39] and Covidence [40] (a systematic review manager) to identify and remove duplicates. Just prior to publication, a search update was conducted on the 15 January 2025 to identify new literature published since the original search date. The full search strategy is available in the Supplementary Material.

### Study Selection

All title and abstracts were reviewed in Covidence using the eligibility criteria by at least two researchers independently (TR, ED, MK). No papers needed translating and all full texts were obtained through either online or inter-library loan. Full-text screening and data extraction was performed by two reviewers (TR, MK) to ensure accuracy and completeness. Basic information was extracted from included studies using an adapted JBI checklist added into the Covidence template [40]. Conflicts were resolved by the senior author (JS) in deliberation with the two lead reviewers. The search update was independently dual screened (JS, ED) with conflicts resolved through discussion.

### Data Extraction

Data extracted in the Covidence template included general article information, including title, journal, country of origin, year of publication, and year of data collection, as well as characteristics of the study, such as study design, aims, setting, population, eligibility criteria, data collection method, and sample size. Data such as counts, means, and rates reported in the published papers and any supplementary materials were extracted and entered in an Excel spreadsheet.

### Synthesis Methods

The extracted data were imported into RevMan Web [41] to generate pooled odds ratios or mean differences, and their associated forest plots. Chi^2^ and I^2^ statistic were calculated and used as an indicator for heterogeneity of the studies. An I^2^ statistic less than 30% was considered to represent low heterogeneity [42].

### Assessment of Risk of Bias

The JBI critical appraisal tools for cross sectional, cohort and case control studies (2020 version) were used to assess for risk of bias for each study [43]. Two researchers (TR, MK) independently conducted the assessment of bias for each included study using an Excel spreadsheet template. Disagreements were discussed with a third researcher (JS) to reach consensus.

### Assessment of Cultural Appropriateness

Two appraisal tools were used to assess the cultural appropriateness of the reporting of research involving Indigenous people. The CONSolIDated critERia (CONSIDER) [44] can be used to assess the reporting of health research involving Indigenous Peoples and was developed from guidelines from a diverse range of international Indigenous Peoples. The Centre of Research Excellence in Aboriginal Chronic Disease Knowledge Translation and Exchange (CREATE) [45] tool was developed with a focus on the reporting of research involving the Aboriginal and Torres Strait Islander people of Australia. Despite this Australian focus, we used the CREATE tool to assess the involvement of Indigenous people, knowledges, and methodology within all included studies, as the tool is robust and comprehensive. Two researchers (ED, CN) used both appraisal tools to independently assess the included studies using an Excel spreadsheet template. Interrater reliability was calculated. Disagreements in assessment were discussed and a third researcher (JS) involved in the consensus decision making process.

### Researcher Reflexivity

The authors identify as Indigenous and non-Indigenous women. The first author (JS) is a non-Indigenous epidemiologist with experience conducting mixed methods research. Four authors (ED, TR, MK, CN) are non-Indigenous researchers with varied experience working in Indigenous settings. Three authors (TJ, JS, CR) are Aboriginal researchers and leaders in their communities.

## Results

### Study Selection

**Fig. 1:**
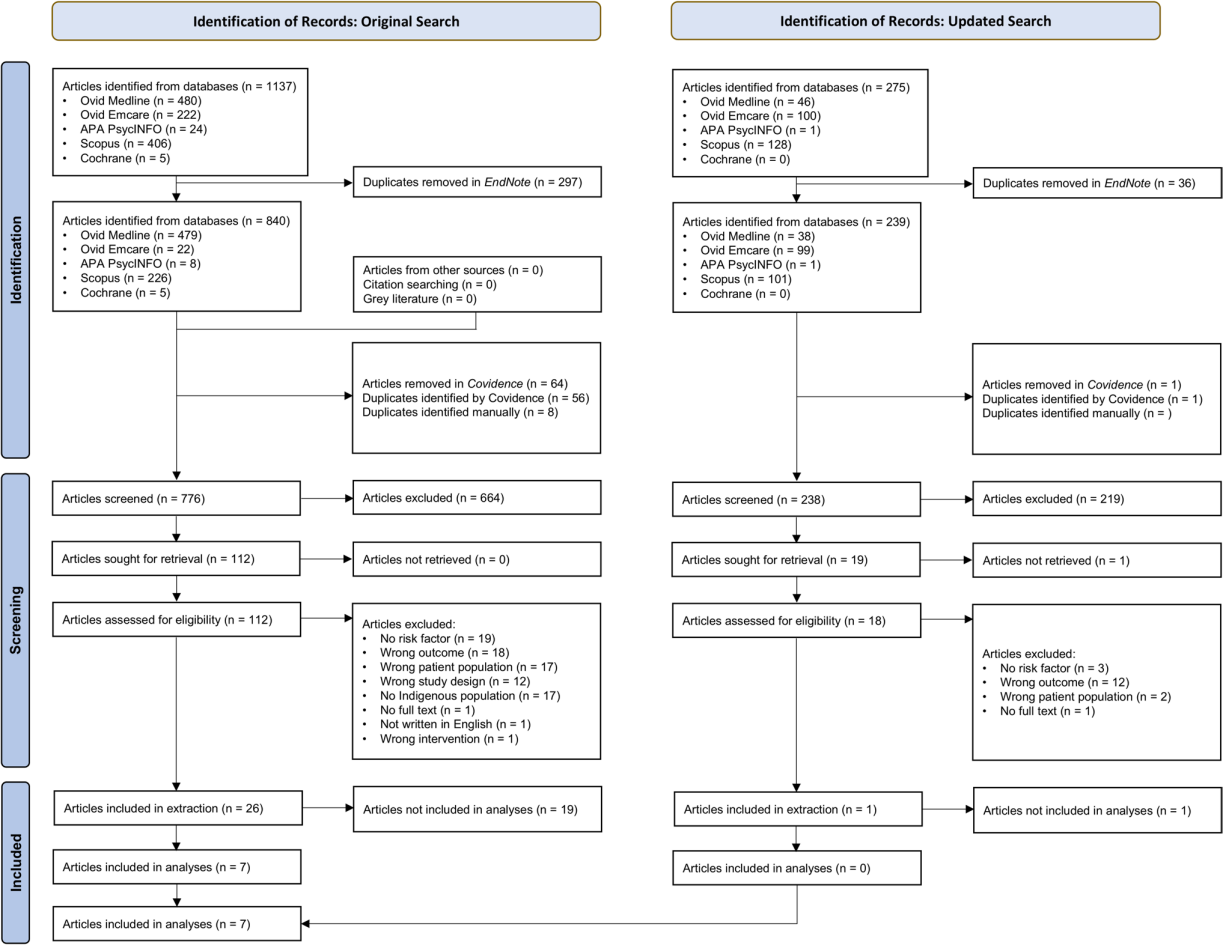
Article identification and selection process: PRISMA Flowchart

The original search yielded 1,137 papers in which 361 duplicates were identified and removed. Title and abstract screening of the remaining 776 papers identified 112 papers for full text screening. Twenty-six papers were identified for inclusion and underwent data extraction, however further investigation of the extracted data showed eighteen papers needed to be excluded. The main reasons for exclusion were the data were not extractable or the article reported risk factors not reported in other papers. Three papers reported findings from the same study from overlapping time periods, with the same risk factors reported in each paper; except one paper which reported an additional risk factor. Of these three papers, two were included: the paper with the largest sample size, and paper with the additional risk factor (although only the additional risk factor data was extracted). The search update identified 238 additional papers, with one possible study identified for inclusion; however, data were not extractable, and the paper was excluded. Therefore, seven papers (reporting data from six studies) were included in the meta-analysis (Fig. 1).

### Study Characteristics

The six studies [20, 46–51] included in the final data analysis were: four cohort studies, one case control study, and one with cohort and case control phases (two published papers). The papers were published between 2005 and 2018 with data collected between 1994 and 2014. Four studies were conducted in Australia [20, 49–51], one in Canada, and one in the United States (two papers from the same study but different time periods) [46, 47]. No studies were identified from New Zealand. The Australian studies included Aboriginal and Torres Strait Islander women from Queensland, New South Wales, and Northern Territory [20, 49–51]. The two papers from the United States were from the same lead author but had different data collection periods (December 2004 to August 2009, and December 2004 to March 2008) and included American Indian women from the same community [46, 47]. The one study from Canada included Cree women (an Indigenous Canadian tribe) from Subartic and Plains region [48]. Preeclampsia was the outcome of interest in the study from the United States [46, 47], whereas HDP was the outcome of interest for the Australian and Canadian studies [20, 48–51]. None of the studies differentiated between term and preterm preeclampsia or HDP. Table 1 summarises the characteristics of the included studies.

**Table 1:** Summary of study characteristics

First Author, year	Outcome	Total	Total with PE/HDP	Total without PE/HDP	Country	Type of study	Time of data collection	Data source	Setting	Population description	Inclusion criteria	Exclusion criteria
Best 2009 [46]	PE	252	87	165	United States	Case Control, Cohort	Dec 2004 – Mar 2008	80% from Turtle Mountain database, 20% self-identified	Hospital and clinic	Tribal members of the Turtle Mountain Band of Chippewa.	Developed hypertension, proteinuria and other signs of preeclampsia after 20 weeks gestation	Pre-existing hypertension or proteinuria before pregnancy
Best 2012 [47]	PE	299	101	198	*As above*	*As above*	Dec 2004 – Aug 2009	*As above*	*As above*	*As above*	*As above*	*As above*
Brennand 2005 [48]	HDP	603	58	545	Canada	Cohort	Jan 1994 – Dec 2000	Medical records of Cree women in James Bay	Hospital	Cree women living in James Bay, Quebec	First full-term singleton birth recorded between January 1994 and December 2000. Pregravid BMI >18.5kg/m, weight recorded in first trimester and within one month prior to delivery.	Pregnancies with factors that may have influenced maternal weight gain. First and final weights in pregnancy recorded outside time range. Glycemic abnormalities before pregnancy.
Campbell 2013 [49]	HDP	220	22	198	Australia	Cohort	Jun 2009 – Sept 2009	1998-2000 Health screening program combined with 1998-2008 Queensland hospitalisation and perinatal data.	Hospital	Aboriginal and Torres Strait Islander women from 26 rural and remote communities in Queensland.	Women hospitalised for birth of infant at least 20 weeks or birthweight >400g.	Pregnant at time of their health check.
Daly 2018 [20]	HDP	85	31	54	Australia	Cohort	Jul 2014 – Aug 2014	Clinic patients	Tharawal Aboriginal Medical Service	Aboriginal and Torres Strait Islander women 18-45 years.	Female, previously given birth, currently 18-45 years, not pregnant currently and identified as Aboriginal or Torres Strait Islander.	Not Aboriginal or Torres Strait Islander, not in specified age range or currently pregnant.
Sina 2014 [50]	HDP	168	25	143	Australia	Case Control	2012	Health Screening program	Hospital	Women from one tribal group in the Northern Territory of Australia	Women who become pregnant during follow up period.	Women that were pregnant before baseline anthropometric measurements. Missing baseline measurements (BMI, WHR, HC).
Thrift 2014 [51]	HDP	13,582	696	12,886	Australia	Cohort	1 Jul 2007 – 31 Dec 2011	Queensland Perinatal Data Collection	Hospital and non-hospital	All hospital and non-hospital births in Queensland of at least 20 weeks' gestation or at least 400g birthweight.	Singleton, at least 20 weeks or 400g birthweight.	Multiple births, gestations less than 20 weeks or 400g birthweight. Missing maternal data.

Two studies were able to provide data for the meta-analyses of age, alcohol consumption, systolic blood pressure (SBP), diastolic blood pressure (DBP), pre-pregnancy diabetes (type unknown), gestational diabetes, and high microalbuminuria. Three studies provided data for the meta-analyses of body mass index (BMI), overweight and obesity (according to BMI), and smoking. Overweight and obese categories were compared to healthy weight (BMI between 18.5 and 24.9 kg/m^2^). Several factors associated with preeclampsia were not able to be included in the meta-analysis because only one study reported the results: education level, parity, multiple births, waist-circumference, weight gain in pregnancy, genetic markers, fruit and vegetable intake, physical activity, cholesterol and lipids, and health service usage or antenatal care.

### Main Findings

**Fig. 2:**
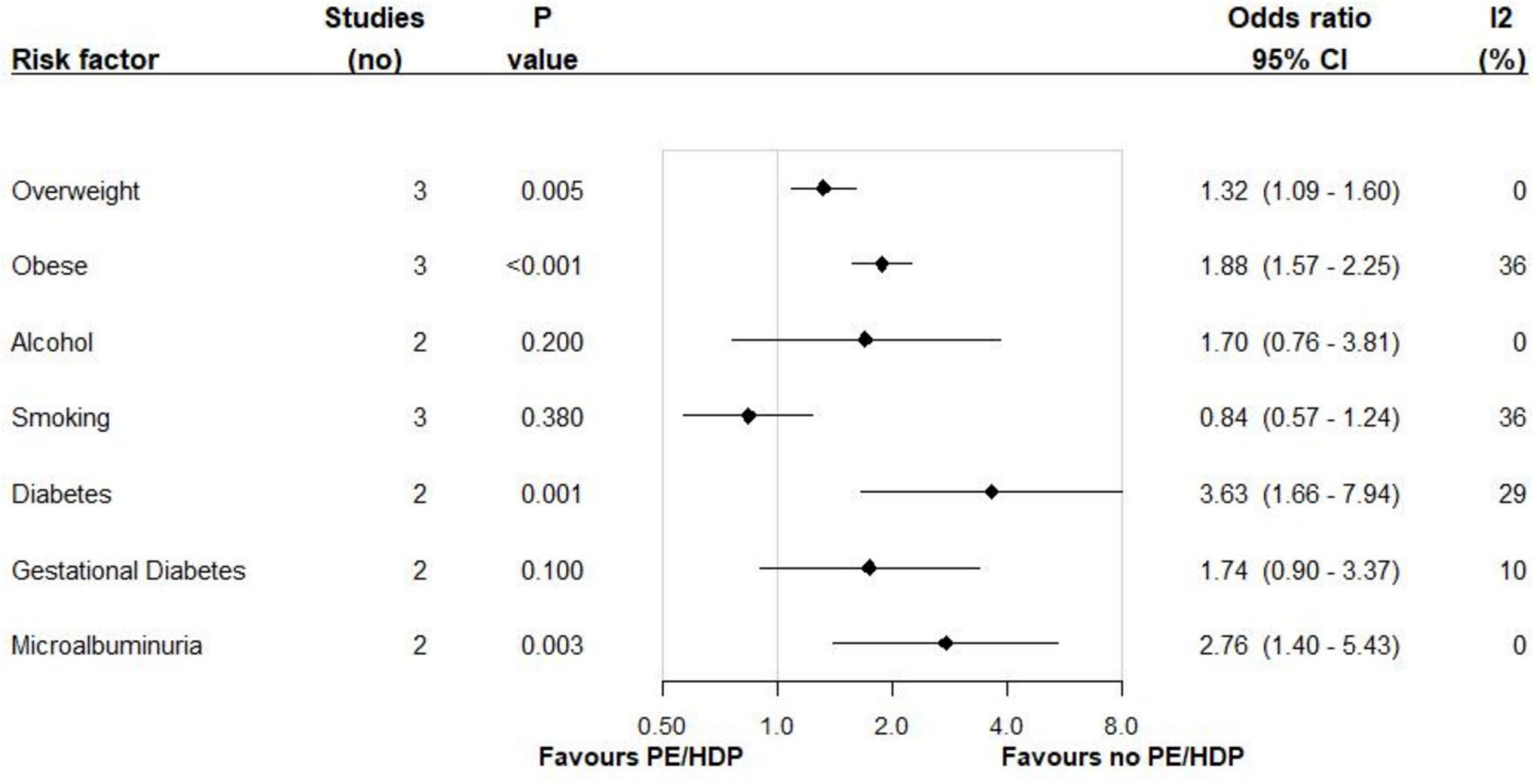
Summary of the associations between preeclampsia/Hypertension Disorders in Pregnancy and various potential factors among Indigenous women. Footnote: CI: confidence interval; I2: I^2^; PE: Pre-eclampsia; HDP: Hypertension Disorders in Pregnancy; Overweight and Obese classifications are compared to normal weight as classified by Body Mass Index

**Fig. 3:**
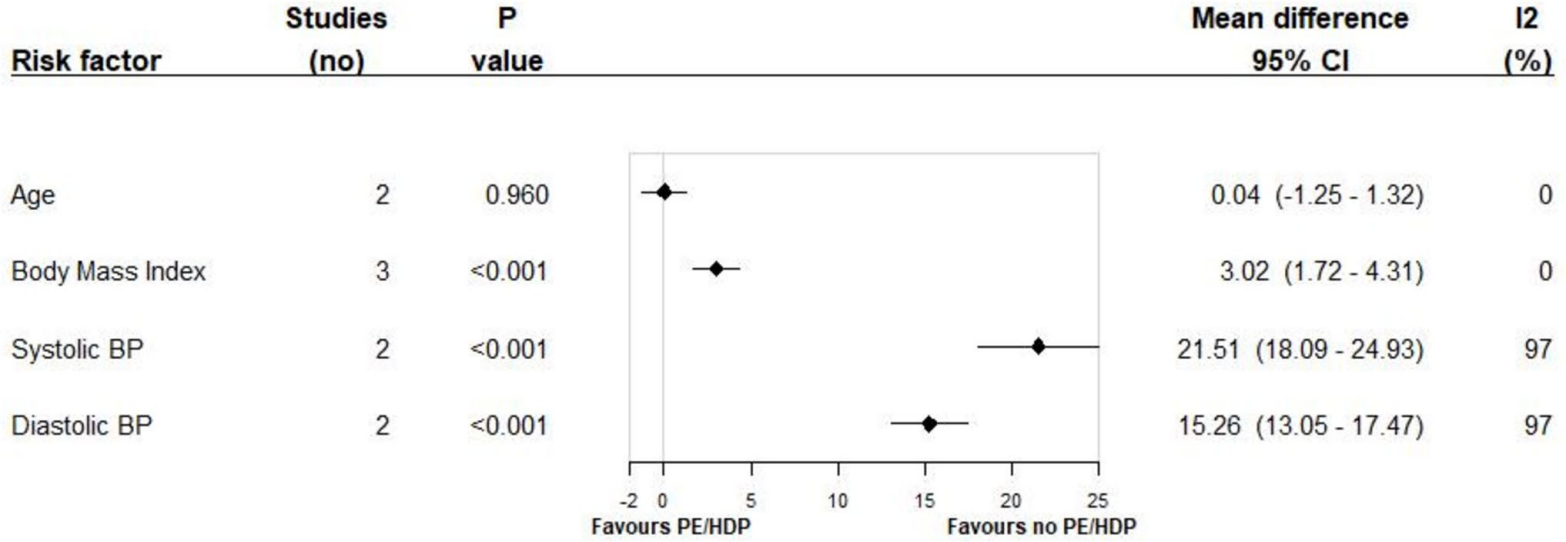
Summary of pooled mean differences between preeclampsia/Hypertension Disorders in Pregnancy and various factors among Indigenous women. Footnote: CI: confidence interval; I2: I2; PE: Pre-eclampsia; HDP: Hypertension Disorders in Pregnancy; BMI: Body Mass Index; BP: Blood Pressure

Figures 2 and 3 show the weighted pooled odds ratios and mean differences for preeclampsia/HDP for each risk factor for Indigenous women. Additional forest plots are available in the Supplementary Materials. Indigenous women who had preeclampsia/HDP were more likely to be classified as overweight (OR 1.32, 95% CI: 1.09–1.60) or as obese (OR 1.88, 95% CI: 1.57–2.25), having pre-pregnancy diabetes (OR 3.63, 95% CI: 1.66–17.94), having high microalbuminuria (OR 2.76, 95% CI: 1.40–5.43), higher mean BMI (MD 3.02 95% CI: 1.72–4.31), and higher mean SBP (MD 15.19 95% CI: 12.83–17.541) and DBP (MD 15.26 95% CI: 13.05–17.47). There was no (or weak) association found between preeclampsia/HDP and mean age (MD 0.04 95% CI: −1.25–1.32), alcohol consumption (OR 1.70, 95% CI: 0.76–3.81), smoking (OR 0.84, 95% CI: 0.57–1.24), and gestational diabetes (OR 1.74, 95% CI: 0.90–3.37). Low heterogeneity (I^2^ statistic) was observed for age, overweight, BMI, alcohol consumption, pre-pregnancy diabetes, gestational diabetes, and microalbuminuria. However, substantial heterogeneity was observed for mean SBP and DBP and moderate heterogeneity was observed for obesity and smoking, making the outcomes of these meta-analyses unreliable.

### Assessment of Risk of Bias

The results of the critical appraisal using JBI tools [43] are reported in Table 2.. Cohort and case control studies are tabled separately, as questions were slightly different within the method-specific tools. All eight papers were included in the meta-analysis after going through critical appraisal. In general, the papers were reported, and the studies conducted, using methods that reduce bias.

**Table 2:** Critical appraisal scores for the included studies: **a**) cohort studies, **b**) case control studies

	1. Were the two groups similar and recruited from the same population?	2. Were the exposures measured similarly to assign people to both exposed and unexposed groups?	3. Was the exposure measured in a valid and reliable way?	4. Were confounding factors identified?	5. Were strategies to deal with confounding factors stated?	6. Were the groups/participants free of the outcome at the start of the study (or at the moment of exposure)?	7. Were the outcomes measured in a valid and reliable way?	8. Was the follow up time reported and sufficient to be long enough for outcomes to occur?	9. Was follow up complete, and if not, were the reasons to loss to follow up described and explored?	10. Were strategies to address incomplete follow up utilized?	11. Was appropriate statistical analysis used?	
Best 2009 [46]									NA	NA		Include
Best 2012 [47]									NA	NA		Include
Brennand 2005 [48]									NA	NA		Include
Campbell 2013[49]									NA	NA		Include
Daly 2018 [20]									NA	NA		Include
Thrift 2014 [51]									NA	NA		Include

**Table:** 

	1. Were the groups comparable other than the presence of disease in cases or the absence of disease in controls?	2. Were cases and controls matched appropriately?	3. Were the same criteria used for identification of cases and controls?	4. Was exposure measured in a standard, valid and reliable way?	5. Was exposure measured in the same way for cases and controls?	6. Were confounding factors identified?	7. Were strategies to deal with confounding factors stated?	8. Were outcomes assessed in a standard, valid and reliable way for cases and controls?	9. Was the exposure period of interest long enough to be meaningful?	10. Was appropriate statistical analysis used?	
Best 2009 [46]											Include
Best 2012 [47]											Include
Sina 2014 [50]											Include

Key:  Yes;  No;  Unclear; *NA* Not applicable

### Assessment of Cultural Appropriateness

The seven included studies were assessed as having a very low level of culturally appropriate involvement of Indigenous People in the conduct and reporting of their research. There was a substantial level of agreement between the two researchers when using the CONSIDER criteria (IRR 67.9%, 95% CI: 54.7–84.0) and a moderate level of agreement when using the CREATE tool (42.59%, 95% CI 27.8–55.2) for the assessment of cultural appropriateness.

When assessed with the CONSIDER statement, one met five (29.41%) criteria, one met three (17.65%) criteria, three met two (11.76%) criteria and two met only one (5.88%) criteria. While one criteria was met by all the studies (Criteria 4 which requires studies to explain how the study aims emerged from priorities identified by Indigenous Peoples), 10 of the 17 items (58.82%) of the CONSIDER Statement were not achieved by any study. Specifically, these items related to governance, methodologies, participation, capacity development, and analysis. Approximately half (n = 4, 57.14%) of the studies specified how consent was obtained from Indigenous Peoples for access to data for future research purposes. The studies reporting items in the CONSIDER statement are presented in Supplementary Material 3, Table 1.

When assessed with the CREATE checklist tool, four (57.14%) studies did not meet any of the criteria. One study met three (17.65%), one study met two (11.76%), and another met one (5.88%) of the CREATE criteria. Most (71.42%) of the CREATE criteria (10 of the 14 criteria) were not met by any of the studies. The studies reporting items in the CREATE checklist are presented in Supplementary Material 3, Table 2..

## Discussion

This is the first systematic review and meta-analysis of factors associated with pre-eclampsia/HDP among Indigenous women. Our searches identified six studies (published across seven papers) which reported eleven factors. This meta-analysis found Indigenous women who were overweight or obese, or who had pre-pregnancy diabetes, high blood pressure, or high microalbuminuria, were at increased risk of preeclampsia/HDP during pregnancy. However, this meta-analysis was unable to determine an association between preeclampsia/HDP and age, alcohol consumption, smoking, and gestational diabetes. In general, the findings reported herein are consistent with other meta-analyses of the factors associated with the development of preeclampsia/HDP amongst non-Indigenous pregnant women [10, 13, 14, 52–62].

The meta-analysis found overweight Indigenous women had a slightly higher odds (OR 1.32, 95% CI: 1.09–1.60) of developing preeclampsia/HDP than women who were not overweight, whereas obese Indigenous women had nearly twice the odds (OR 1.88, 95% CI: 1.57–2.25) of developing preeclampsia/HDP than other women. This finding is similar to extensive evidence from meta-analyses of preeclampsia in the non-Indigenous population which shows obesity in pregnant women increases preeclampsia risk [10, 14, 54, 56–59]. For example, in a meta-analysis of 265,270 births from across 39 European, North American, and Australian cohorts, obesity doubled the risk of preeclampsia [59]. Similarly, the risk of preeclampsia among obese women was estimated as 2.8 times higher than for their counterparts in a global meta-analysis of 92 studies [10].

Our findings demonstrate a strong association between pre-existing diabetes and preeclampsia/HDP amongst Indigenous women (OR 3.63, 95% CI: 1.66–17.94). Similarly, a meta-analyses in the non-Indigenous population, found women with pre-existing type 2 diabetes were 3.7 times more likely to experience preeclampsia during pregnancy [10]. Furthermore, women with type 1 diabetes were 3.6 times more likely to develop preeclampsia/HDP [52]. Furthermore, this review’s findings demonstrate Indigenous women who had microalbuminuria (protein in urine) prior to their pregnancy were nearly three times more likely to develop preeclampsia/HDP (OR 2.76, 95% CI: 1.40–5.43). This is consistent with a review of 11 studies in the non-Indigenous population which found women with microalbuminuria had between 3.8 and 11.7 time the odds of developing preeclampsia during pregnancy [60].

The association between pre-existing diabetes, microalbuminuria and preeclampsia/HDP is notable for Indigenous women, as it has been well documented Indigenous women are disproportionately impacted by high rates of diabetes and kidney disease compared to the non-Indigenous population [8, 24, 63]. Furthermore, evidence suggests there are increasing rates of diabetes and kidney disease amongst Indigenous women [63], and thus potential increased burden of preeclampsia – as well as long term chronic health outcomes. As such, there is a clear need to ensure clinicians are aware of the link between pre-existing diabetes, microalbuminuria and preeclampsia/HDP to ensure appropriate early detection, monitoring and treatment can be implemented.

Preexisting hypertension (mean SBP and DBP) was associated with preeclampsia/HDP amongst Indigenous women. However, of the included studies, one used blood pressure recorded prior to pregnancy [50] while the other reported measurements taken after 20 weeks gestation [47], resulting in large variations in the reported measurements. Furthermore, the sample size for one study [47] was 70% larger, which skewed the findings towards a positive association. As such, more data is needed to improve confidence in this finding, that there is an association between preexisting hypertension and preeclampsia/HDP for Indigenous women. However, previous meta-analyses show the non-Indigenous woman with preconceptual hypertension have between 2.5 to 5.1 times the risk of developing preeclampsia than their non-hypertensive counterparts [10, 55, 60]. As such, clinicians recommend optimal care for women with pre-existing hypertension should include pre-conceptual counselling, frequent antenatal visits and monitoring of blood pressure during pregnancy, as well as peripartum and postpartum monitoring and follow up [64].

Importantly, it should be noted these factors and pre-existing conditions are also closely interrelated with similar pathophysiological profiles [65]. Obesity, diabetes, and high blood pressure are all part of the larger constellation of conditions that together are termed ‘metabolic syndrome’ and which increase the risks of heart disease, stroke, and type 2 diabetes [66]. Women with metabolic syndrome are at an increased risk of preeclampsia (RR 1.63, 95% CI: 1.23 to 2.15 [67] and OR 1.48, 95% CI: 1.26—1.74 [68]). Insulin resistance – another component of metabolic syndrome – also plays a role, with women who develop preeclampsia more likely to be insulin resistant compared to normotensive women [65]. While evidence suggests preeclampsia incidence is increased in women with gestational diabetes, it is unclear if gestational diabetes is independently related to the occurrence of preeclampsia [65]. As a result of the interrelatedness of these metabolic syndrome components, it can be difficult to clearly determine independent associations. Despite this, it is clear women with pre-existing cardiovascular conditions are at higher risk of preeclampsia/HDP.

In contrast, findings on associations between alcohol and preeclampsia were not consistent with current evidence, which suggested a possible link despite a wide confidence interval (OR 1.70, 95% CI 0.76–3.81). A global systematic review of 37 studies of over four million women found no association between alcohol consumption during pregnancy and preeclampsia (OR = 0.93, 95%CI: 0.73–1.20) [13]. Finally, our finding on the association between smoking and preeclampsia risk was consistent with current literature, which suggests a spurious inverse relationship between smoking and preeclampsia risk [14, 53, 61]. The most likely explanation for this illogical inverse relationship, which suggests smoking is preventive of preeclampsia/HDP, is natural selection bias due to a loss of women and pregnancies from study populations [69].

### Strengths and Limitations

This systematic review followed a rigorous systematic process. Despite this, there are several limitations which need to be considered when drawing conclusions from the evidence. Firstly, the aim of this systematic review was to review the evidence for Indigenous women over a range of similarly colonised countries. However, the identified evidence was sparse, with only one study from Canada and the United States, and no studies from New Zealand, identified for inclusion. As a result, the findings reported are skewed towards Aboriginal and Torres Strait Islanders women and may not be generalisable to the diversity of Indigenous women or Indigenous women globally. Furthermore, differences in how each country recognises their Indigenous Populations and how race/ethnicity is socially, culturally, and politically constructed and reported can also impact on the evidence [70].

Moreover, the assessment of cultural appropriateness found the included studies had a very low level of culturally appropriate research practices. There was a stark lack of Indigenous peoples’ involvement in the governance, methods and conduct, analysis, and reporting of the reported research. The absence of Indigenous people in the conduct of these included studies means the original results were typically reported without context as to why the reported factors were important for the study populations. The studies did not include Indigenous Knowledges or Indigenous Methods in their research methodology [71, 72], and did not acknowledge Indigenous Peoples ways of ‘Knowing, Being and Doing’ [73], nor the ongoing impacts of colonisation, racism, and social injustice [24]. Only half the studies mentioned how consent would be obtained for access to Indigenous data for future research purposes. The inclusion of Indigenous Peoples in research process is important as Indigenous data sovereignty and governance frameworks ensure Indigenous Peoples have input into the “*appropriate presentation of race and ethnicity in health-related data*” [72] thus avoiding the perpetuation of deficient discourses and contributing to the disruption of systemic racism and ongoing power dynamics which continue to be embedded in health research. Research must bring together western academic methods with Indigenous Knowledges, with researchers working at the knowledge interface to ensure health research prioritises Indigenous Peoples [74].

An additional limitation was the definition and diagnosis criteria for preeclampsia/HDP may vary between geographical locations and change over time [2]. Gestational hypertension, preeclampsia, and other HDP-related pregnancy outcomes should be examined separately, however they are often examined in combination despite different diagnostic criteria applying to each condition [75]. Definitions, diagnosis criteria, measurement, and reporting of the factors reported were also susceptible to differences between studies, including when (pre- or peri-natal) and how (self-reported or validated) the data was measured. These variations have likely contributed to a moderate level of heterogeneity and lower effect sizes for some of our estimates, particularly for those where a limited number of studies reported on the factors of interest.

Finally, a limited number of factors were able to be explored in this review due to a paucity of data reported for some factors making meta-analysis not possible. Examples of this were education attainment, parity, multiple pregnancy, disability, and air pollution. No studies were found on health service usage or other socio-demographic indicators, such as income. This demonstrates gaps in the literature on the impact of these factors for the study population.

### Implications for Practice

Indigenous women experience higher rates of preeclampsia, it is important to understand the factors associated with these higher rates. Importantly, these associated factors can be used to identify and prioritise Indigenous women at risk of preeclampsia for prevention, early detection, and treatment during the antenatal period. Furthermore, the identification of associated factors, such as obesity and diabetes, presents an opportunity to integrate pre-pregnancy education programs or preconception care (PCC) into community-led programs to reduce these factors and their impact across the life-course of these women and their children [76, 77]. Taking a life-course approach to preeclampsia prevention among young women is essential given the increased and interlinked rates of obesity, diabetes, and other chronic cardiovascular conditions, which has become increasingly evident over the last 30 years and which contribute to the increased burden of disease among Indigenous communities. As such, healthcare providers must integrate education on preeclampsia, its links to other conditions, and its potential impact on increased risk of developing cardiovascular conditions in later life, into a broader chronic disease prevention, management, and care program. Furthermore, screening tools used for Indigenous women’s maternal and infant care programs must be updated to ensure women’s pre-disposition to developing pre-eclampsia and HDP is identified and actioned early in their care plans.

Health practitioners must be made aware of the links between these factors and preeclampsia to enable them to provide better support and care to Indigenous women during family planning and antenatal discussions. Guidelines recommend increased antenatal care and early visits in pregnancy to reduce severe complications to mother and baby due to preeclampsia [2]. Despite this, engagement in prenatal care has been consistently lower amongst Indigenous women [28, 30]. A potential contributor to this lower engagement in antenatal care may be that currently available antenatal care is not culturally appropriate or safe. Indigenous women who having a strong connection to traditional lands and community have been shown to have better maternal and neonatal outcomes [78, 79]. Antenatal services which are community-led and delivered can improve engagement and clinic outcomes [78]. The provision of culturally safe, quality antenatal services for Indigenous women influences antenatal engagement, having positive experiences, reducing fear and anxiety in accessing services, and improved health outcomes for both mother and babies [78, 80–83]. Moreover, a ‘Birthing on Country’ model of care has been shown to be a cost-effective alternative to standard care [84], reducing preterm birth [78, 84], and result in less postnatal neonatal admissions [84]. Therefore, a culturally safe prevention program to address key factors, such as obesity and diabetes, for women during the preconception period must include improving community infrastructure and access to services [85].

## Conclusion

Preeclampsia is a common hypertensive condition encountered during pregnancy [19]. Although a well-recognised pregnancy disorder, there are still gaps and inconsistencies in knowledge related to factors, particularly for some population groups including Indigenous women from Canada, Australia, New Zealand and the United States. Through the systematic review process, it was found that obesity/overweight, pre-pregnancy diabetes, and microalbuminuria are associated with preeclampsia in Indigenous women. Smoking was not found to be associated with preeclampsia in Indigenous women, although this is similar to other studies amongst non-Indigenous population. Identifying factors associated with increased preeclampsia risk amongst Indigenous and Indigenous women is important to minimising adverse perinatal events and future health complications. Although, this review has not made it clear as to whether alcohol consumption and smoking is a risk factor for preeclampsia or HDP, this review demonstrates there is currently limited data related to pregnancy outcomes of Indigenous women, particularly in New Zealand and Canada. Further culturally appropriate work is needed in this area, specifically in relation to social, economic, and environmental factors.

## Key References

- Chappell LC, Cluver CA, Kingdom J, Tong S. Pre-eclampsia. Lancet. 2021;398(10297):341–354.
  - ○ This paper provides a comprehensive overview of the current evidence on the pathogenesis, epidemiology, prevention, screening, treatment options, and long-term health impact on women who experience preeclampsia.
- Smallwood R, Woods C, Power T, Usher K. Understanding the Impact of Historical Trauma Due to Colonization on the Health and Well-Being of Indigenous Young Peoples: A Systematic Scoping Review. J Transcult Nurs. 2021;32(1):59–68.
  - ○ This paper undertook a scoping review of how historical trauma have had on the health and health disparities in Indigenous populations in countries who have similar colonisation experiences (Canada, Australia, New Zealand and the United States). This paper provides the context of why our study needs to be undertaken.
- Gao Y, Roe Y, Hickey S, et al. Birthing on country service compared to standard care for First Nations Australians: a cost-effectiveness analysis from a health system perspective. Lancet Reg Health West Pac. 2023; 34:100,722.
  - ○ This paper demonstrated a culturally appropriate birthing service offered a cost-effective alternative to standard care in reducing preterm birth for Australian First Nations families.

## Supplementary Information

Below is the link to the electronic supplementary material.

Supplementary file1 (DOCX 67 KB)

Supplementary file2 (DOCX 377 KB)

Supplementary file3 (DOCX 23 KB)

## Data Availability

No datasets were generated or analysed during the current study.
