# Supplementary material for: Factors associated with preeclampsia and the hypertensive disorders of pregnancy amongst Indigenous women of Canada, Australia, New Zealand, and the United States: A systematic review and meta-analysis
Source: Curr Hypertens Rep. 2025 Feb 20;27(1):10. doi: 10.1007/s11906-025-01327-6 (PMC11842517; doi:10.1007/s11906-025-01327-6)
Supplement: Supplementary file 1 — Supplementary file1 (DOCX 67 KB) [file 11906_2025_1327_MOESM1_ESM.docx]

# Supplementary Materials 1: Literature Search Strategy

Key:

| / | MeSH Subject Heading |
| --- | --- |
| * | Truncation |
| mp | Keyword |
| adjn | Proximity |
| $ | Truncation |
| ? | Wildcard |
| W/n | Proximity |
| NEAR/n | Proximity |

Ovid Medline

| 1 | Indigenous Peoples/ or american native continental ancestry group/ or indians, north american/ or alaskan natives/ or indigenous canadians/ or inuits/ or oceanic ancestry group/ |
| --- | --- |
| 2 | Health Services, Indigenous/ |
| 3 | (Aboriginal* or Torres Strait Islander or ATSI).mp. |
| 4 | (Kaurna or Adnyamathanha or Mula or Maralinga or Narungga or Ngaanyatjarra or Ngarrindjeri or Pitjantjatjara or Yolngu or Anangu or Yankunytjatjara or Arrernte or Aranda or Arunta or Arrarnta).mp. |
| 5 | (Inuit* or Inupiat* or Metis* or Metis or Kalaallit* or aleut* or eskimo* or Whenua* or Iwi*).mp. |
| 6 | (Maori* or Maori or "pacific islander*" or Samoan or Fijian or Polynesian).mp. |
| 7 | (indigenous or "first nation*" or "first people*").mp. |
| 8 | (Native American* or American Indian*).mp. |
| 9 | (Innu or Montagnais or Naskapi or Cree or Ojibwa or Chipewyan or Beaver or Slave or Carrier or Gwichin or Tanaina or Deg Xinag or Algonquin or Iroquois or Huron or Wampaoag or Mohican or Mohegan or Ho chunk or Sauk or Fox or Illinois or Cherokee or Chocteaw or Chickasaw or Creek or Seminole or Natchez or Caddo or Apalachee or Timucua or Guale or Mandan or Hidatsa or Omaha or Pawnee or Arikara or Sioux or Blackfoot or Cheyenne or Comanche or Arapaho or Kiowa or Pueblo Indian or Zuni or Hopi or Yuman or Pima or Tohono Oodham or Papago or Navajo or Apache or Washoe or Mono or Paiute or Bannock or Shoshone or Ute or Gosiute or Hupa or Yurok or Pomo or Yuki or Wintan or Maidu or Yana or Tlingit or Haida or Tsimshian or Kwakiutl or Bella Coola or Nuu chah nulth or Nootka or Coast Salish or Chinook or Salish or Flathead or Nez Perce or Yakama or Modoc or Klamath or Spokan or Kalispel or Pend dOreille or Coeur dAlene or Walla Walla or Umatilla).mp. |
| 10 | or/1-9 |
| 11 | Pre-Eclampsia/ |
| 12 | Hypertension, Pregnancy-Induced/ |
| 13 | Eclampsia/ |
| 14 | HELLP Syndrome/ |
| 15 | ((edema or oedema or proteinuria or hypertension or hypertensive) adj5 (gestosis or gestoses or antenatal or prenatal or gestation* or perinatal)).mp. |
| 16 | Eclamp*.mp. |
| 17 | HELLP.mp. |
| 18 | (pre-eclampsia or pre eclampsia OR preeclampsia).mp. |
| 19 | (tox?emia ADJ2 (eph or pregnan*)).mp. |
| 20 | (eph adj (complex OR gestosis OR tox?emia*)).mp. |
| 21 | or/11-20 |
| 22 | 10 and 21 |

Ovid Emcare

| 1 | Indigenous Peoples/ or american native continental ancestry group/ or indians, north american/ or alaskan natives/ or indigenous canadians/ or inuits/ or oceanic ancestry group/ |
| --- | --- |
| 2 | Health Services, Indigenous/ |
| 3 | (Aboriginal* or Torres Strait Islander or ATSI).mp. |
| 4 | (Kaurna or Adnyamathanha or Mula or Maralinga or Narungga or Ngaanyatjarra or Ngarrindjeri or Pitjantjatjara or Yolngu or Anangu or Yankunytjatjara or Arrernte or Aranda or Arunta or Arrarnta).mp. |
| 5 | (Inuit* or Inupiat* or Metis* or Metis or Kalaallit* or aleut* or eskimo* or Whenua* or Iwi*).mp. |
| 6 | (Maori* or Maori or "pacific islander*" or Samoan or Fijian or Polynesian).mp. |
| 7 | (indigenous or "first nation*" or "first people*").mp. |
| 8 | (Native American* or American Indian*).mp. |
| 9 | (Innu or Montagnais or Naskapi or Cree or Ojibwa or Chipewyan or Beaver or Slave or Carrier or Gwichin or Tanaina or Deg Xinag or Algonquin or Iroquois or Huron or Wampaoag or Mohican or Mohegan or Ho chunk or Sauk or Fox or Illinois or Cherokee or Chocteaw or Chickasaw or Creek or Seminole or Natchez or Caddo or Apalachee or Timucua or Guale or Mandan or Hidatsa or Omaha or Pawnee or Arikara or Sioux or Blackfoot or Cheyenne or Comanche or Arapaho or Kiowa or Pueblo Indian or Zuni or Hopi or Yuman or Pima or Tohono Oodham or Papago or Navajo or Apache or Washoe or Mono or Paiute or Bannock or Shoshone or Ute or Gosiute or Hupa or Yurok or Pomo or Yuki or Wintan or Maidu or Yana or Tlingit or Haida or Tsimshian or Kwakiutl or Bella Coola or Nuu chah nulth or Nootka or Coast Salish or Chinook or Salish or Flathead or Nez Perce or Yakama or Modoc or Klamath or Spokan or Kalispel or Pend dOreille or Coeur dAlene or Walla Walla or Umatilla).mp. |
| 10 | or/1-9 |
| 11 | Pre-Eclampsia/ |
| 12 | Hypertension, Pregnancy-Induced/ |
| 13 | Eclampsia/ |
| 14 | HELLP Syndrome/ |
| 15 | ((edema or oedema or proteinuria or hypertension or hypertensive) adj5 (gestosis or gestoses or antenatal or prenatal or gestation* or perinatal)).mp. |
| 16 | Eclamp*.mp. |
| 17 | HELLP.mp. |
| 18 | (pre-eclampsia or pre eclampsia OR preeclampsia).mp. |
| 19 | (tox?emia ADJ2 (eph or pregnan*)).mp. |
| 20 | (eph adj (complex OR gestosis OR tox?emia*)).mp. |
| 21 | or/11-20 |
| 22 | 10 and 21 |

PsycINFO

| 1 | exp Indigenous Populations/ |
| --- | --- |
| 2 | (Aboriginal* or Torres Strait Islander or ATSI).mp. |
| 3 | (Kaurna or Adnyamathanha or Mula or Maralinga or Narungga or Ngaanyatjarra or Ngarrindjeri or Pitjantjatjara or Yolngu or Anangu or Yankunytjatjara or Arrernte or Aranda or Arunta or Arrarnta).mp. |
| 4 | (Inuit* or Inupiat* or Metis* or Metis or Kalaallit* or aleut* or eskimo* or Whenua* or Iwi*).mp. |
| 5 | (Maori* or Maori or "pacific islander*" or Samoan or Fijian or Polynesian).mp. |
| 6 | (indigenous or "first nation*" or "first people*").mp. |
| 7 | (Native American* or American Indian*).mp. |
| 8 | (Innu or Montagnais or Naskapi or Cree or Ojibwa or Chipewyan or Beaver or Slave or Carrier or Gwichin or Tanaina or Deg Xinag or Algonquin or Iroquois or Huron or Wampaoag or Mohican or Mohegan or Ho chunk or Sauk or Fox or Illinois or Cherokee or Chocteaw or Chickasaw or Creek or Seminole or Natchez or Caddo or Apalachee or Timucua or Guale or Mandan or Hidatsa or Omaha or Pawnee or Arikara or Sioux or Blackfoot or Cheyenne or Comanche or Arapaho or Kiowa or Pueblo Indian or Zuni or Hopi or Yuman or Pima or Tohono Oodham or Papago or Navajo or Apache or Washoe or Mono or Paiute or Bannock or Shoshone or Ute or Gosiute or Hupa or Yurok or Pomo or Yuki or Wintan or Maidu or Yana or Tlingit or Haida or Tsimshian or Kwakiutl or Bella Coola or Nuu chah nulth or Nootka or Coast Salish or Chinook or Salish or Flathead or Nez Perce or Yakama or Modoc or Klamath or Spokan or Kalispel or Pend dOreille or Coeur dAlene or Walla Walla or Umatilla).mp. |
| 9 | or/1-8 |
| 10 | Preeclampsia/ |
| 11 | Hypertension/ and Pregnancy/ |
| 12 | HELLP.mp. |
| 13 | ((edema or oedema or proteinuria or hypertension or hypertensive) adj5 (gestosis or gestoses or antenatal or prenatal or gestation* or perinatal)).mp. |
| 14 | Eclamp*.mp. |
| 15 | (pre-eclampsia or pre eclampsia OR preeclampsia).mp. |
| 16 | (tox?emia ADJ2 (eph or pregnan*)).mp. |
| 17 | (eph adj (complex OR gestosis OR tox?emia*)).mp. |
| 18 | or/10-17 |
| 19 | 9 and 18 |

Scopus

(Preeclampsia OR (hypertensi* W/2 pregnan*) OR hellp OR eclampsia OR (tox?emia W/2 pregnan*) OR ((edema OR oedema OR proteinuria OR hypertension OR hypertensive) w/5 (gestosis OR gestoses OR antenatal OR prenatal OR gestation* OR perinatal)))

AND

(Aboriginal* OR “Torres Strait Islander” OR ATSI) OR (Kaurna OR Adnyamathanha OR Mula OR Maralinga OR Narungga OR Ngaanyatjarra OR Ngarrindjeri OR Pitjantjatjara OR Yolngu OR Anangu OR Yankunytjatjara OR Arrernte OR Aranda OR Arunta OR Arrarnta) OR (Inuit* OR Inupiat* OR Metis* OR Metis* OR Kalaallit* OR aleut* OR eskimo* OR Whenua* OR Iwi*) OR (Maori* OR Maori OR "pacific islander*" OR Samoan OR Fijian OR Polynesian) OR (indigenous OR "first nation*" OR "first people*") OR (“Native American*” OR “American Indian*”) OR (Innu OR Montagnais OR Naskapi OR Cree OR Ojibwa OR Chipewyan OR Beaver OR Slave OR Carrier OR Gwichin OR Tanaina OR “Deg Xinag” OR Algonquin OR Iroquois OR Huron OR Wampaoag OR Mohican OR Mohegan OR “Ho chunk” OR Sauk OR Fox OR Illinois OR Cherokee OR Chocteaw OR Chickasaw OR Creek OR Seminole OR Natchez OR Caddo OR Apalachee OR Timucua OR Guale OR Mandan OR Hidatsa OR Omaha OR Pawnee OR Arikara OR Sioux OR Blackfoot OR Cheyenne OR Comanche OR Arapaho OR Kiowa OR “Pueblo Indian” OR Zuni OR Hopi OR Yuman OR Pima OR “Tohono Oodham” OR Papago OR Navajo OR Apache OR Washoe OR Mono OR Paiute OR Bannock OR Shoshone OR Ute OR Gosiute OR Hupa OR Yurok OR Pomo OR Yuki OR Wintan OR Maidu OR Yana OR Tlingit OR Haida OR Tsimshian OR Kwakiutl OR “Bella Coola” OR “Nuu chah nulth” OR Nootka OR “Coast Salish” OR Chinook OR Salish OR Flathead OR “Nez Perce” OR Yakama OR Modoc OR Klamath OR Spokan OR Kalispel OR “Pend dOreille” OR “Coeur dAlene” OR “Walla Walla” OR Umatilla)

Cochrane

(Preeclampsia OR ((hypertensive OR hypertension OR toxaemia OR toxemia) NEAR/2 (pregnant OR pregnancy)) OR hellp OR eclampsia OR ((edema OR oedema OR proteinuria OR hypertension OR hypertensive) NEAR/5 (gestosis OR gestoses OR antenatal OR prenatal OR gestation OR gestantional OR perinatal)))

AND

(Aboriginal OR Aboriginals OR “Torres Strait Islander” OR ATSI OR Kaurna OR Adnyamathanha OR Mula OR Maralinga OR Narungga OR Ngaanyatjarra OR Ngarrindjeri OR Pitjantjatjara OR Yolngu OR Anangu OR Yankunytjatjara OR Arrernte OR Aranda OR Arunta OR Arrarnta OR Inuit OR Inuits OR Inupiat OR Inupiats OR Metis OR Kalaallit OR Kalaallits OR aleut OR aleuts OR eskimo OR eskimos OR Whenua OR Whenuas OR Iwi OR Iwis OR Maori OR Maoris OR "pacific islander" OR “pacific islanders” OR Samoan OR Fijian OR Polynesian OR indigenous OR "first nation" OR “first nations” OR "first people" OR “first peoples” OR “Native American” OR “Native Americans” OR “American Indian” OR “American Indians” OR Innu OR Montagnais OR Naskapi OR Cree OR Ojibwa OR Chipewyan OR Beaver OR Slave OR Carrier OR Gwichin OR Tanaina OR “Deg Xinag” OR Algonquin OR Iroquois OR Huron OR Wampaoag OR Mohican OR Mohegan OR “Ho chunk” OR Sauk OR Fox OR Illinois OR Cherokee OR Chocteaw OR Chickasaw OR Creek OR Seminole OR Natchez OR Caddo OR Apalachee OR Timucua OR Guale OR Mandan OR Hidatsa OR Omaha OR Pawnee OR Arikara OR Sioux OR Blackfoot OR Cheyenne OR Comanche OR Arapaho OR Kiowa OR “Pueblo Indian” OR Zuni OR Hopi OR Yuman OR Pima OR “Tohono Oodham” OR Papago OR Navajo OR Apache OR Washoe OR Mono OR Paiute OR Bannock OR Shoshone OR Ute OR Gosiute OR Hupa OR Yurok OR Pomo OR Yuki OR Wintan OR Maidu OR Yana OR Tlingit OR Haida OR Tsimshian OR Kwakiutl OR “Bella Coola” OR “Nuu chah nulth” OR Nootka OR “Coast Salish” OR Chinook OR Salish OR Flathead OR “Nez Perce” OR Yakama OR Modoc OR Klamath OR Spokan OR Kalispel OR “Pend dOreille” OR “Coeur dAlene” OR “Walla Walla” OR Umatilla)
