# Supplementary material for: Factors associated with preeclampsia and the hypertensive disorders of pregnancy amongst Indigenous women of Canada, Australia, New Zealand, and the United States: A systematic review and meta-analysis
Source: Curr Hypertens Rep. 2025 Feb 20;27(1):10. doi: 10.1007/s11906-025-01327-6 (PMC11842517; doi:10.1007/s11906-025-01327-6)
Supplement: Supplementary file 2 — Supplementary file2 (DOCX 377 KB) [file 11906_2025_1327_MOESM2_ESM.docx]

Figure 2: Risk of Preeclampsia/Hypertension Disorders in Pregnancy among First Nations women with and without various risk factors [might reorder]

| **(a) Age** |
| --- |
| 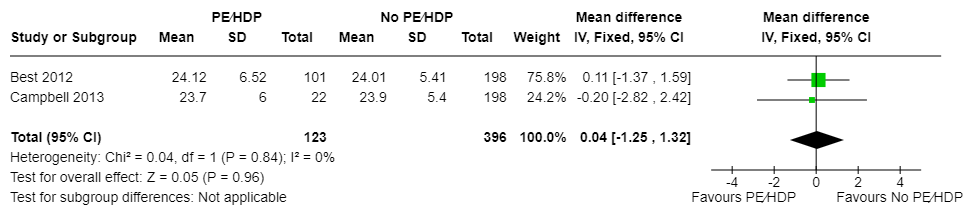 |
| **(b) Overweight (compare to normal)** |
| 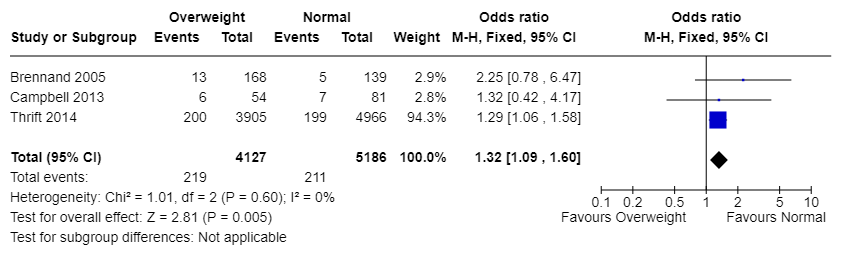 |
| **(c) Obese (compare to normal)** |
| 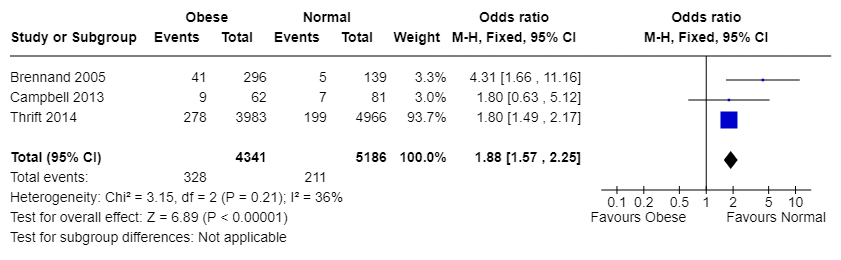 |
| **(d) BMI** |


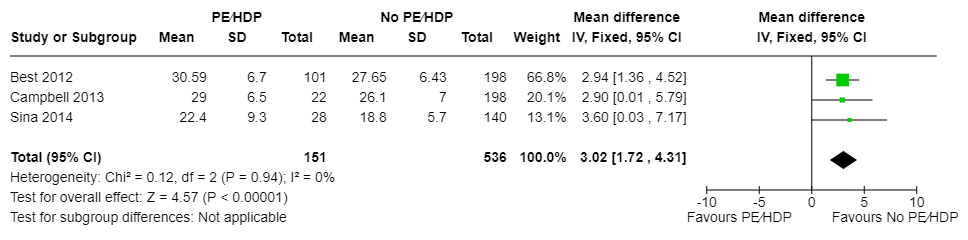


| **(e) Alcohol consumption** |
| --- |
| 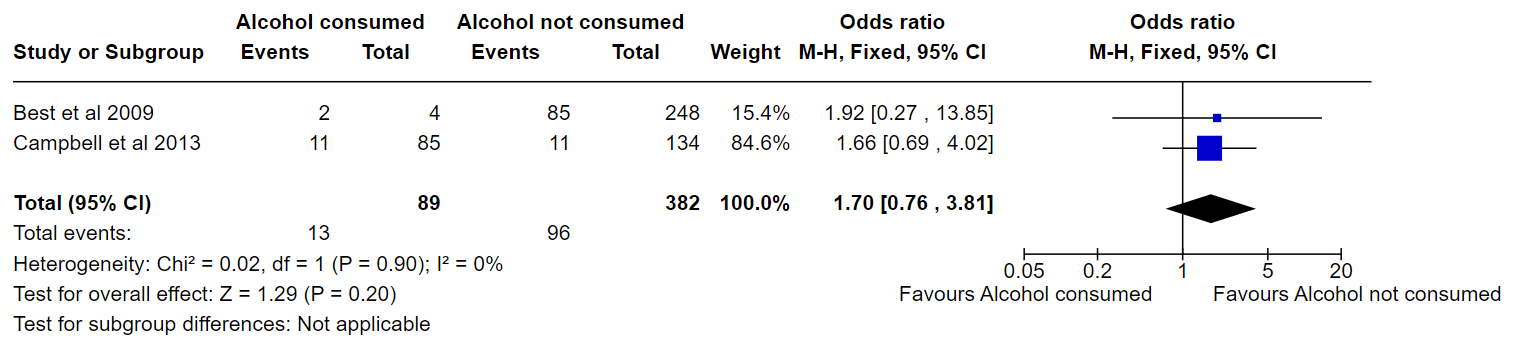 |
| **(f) Smoking** |
| 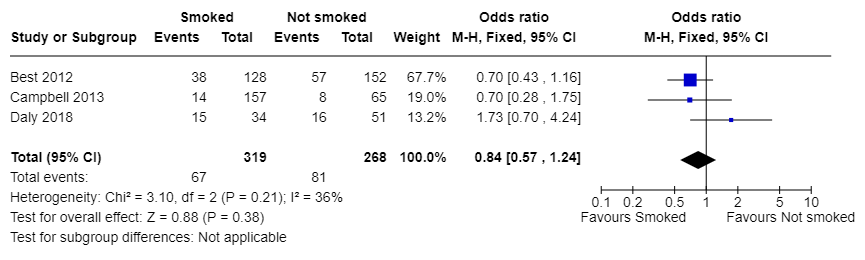 |
| **(g) Systolic Blood Pressure** |
| 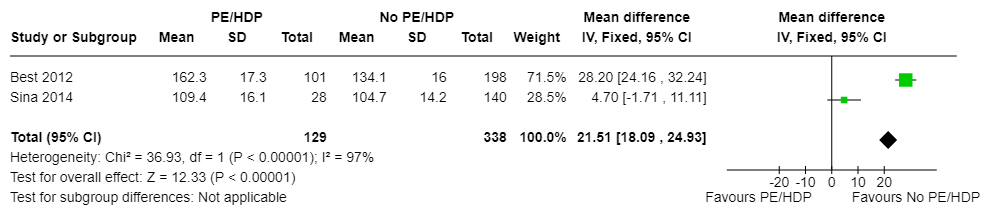 |
| **(h) Diastolic Blood Pressure** |
| 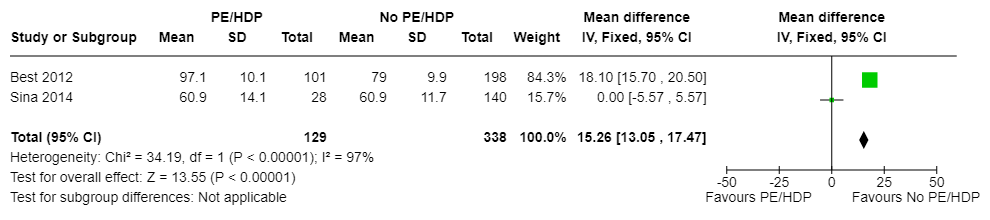 |
| **(i) Diabetes** |
| **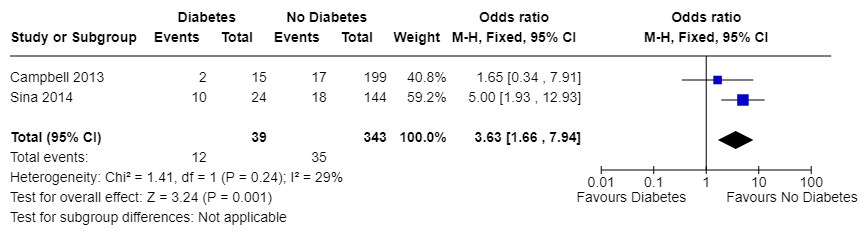** |

| **(j) Gestational Diabetes** |
| --- |
| 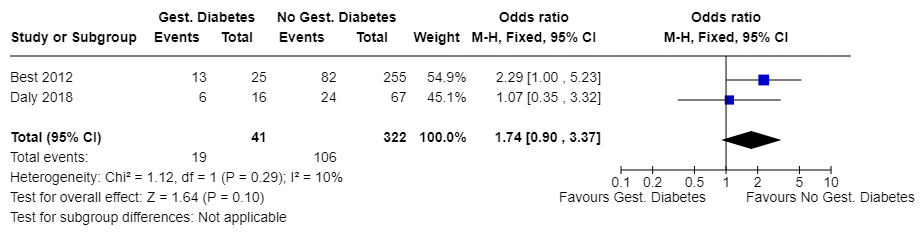 |
| **(k) Micro-albuminuria** |
| **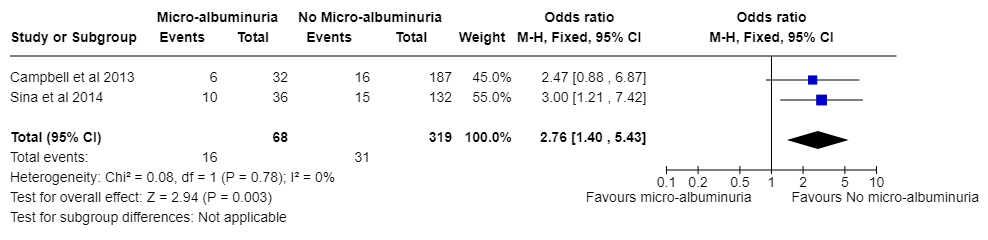** |

Note: HDP Hypertension disorder in pregnancy; PE: Preeclampsia
