# Supplementary material for: Factors associated with preeclampsia and the hypertensive disorders of pregnancy amongst Indigenous women of Canada, Australia, New Zealand, and the United States: A systematic review and meta-analysis
Source: Curr Hypertens Rep. 2025 Feb 20;27(1):10. doi: 10.1007/s11906-025-01327-6 (PMC11842517; doi:10.1007/s11906-025-01327-6)
Supplement: Supplementary file 3 — Supplementary file3 (DOCX 23 KB) [file 11906_2025_1327_MOESM3_ESM.docx]

# SUPPLEMENTARY Material 3, Table 1: Studies Reporting Items in the CONSIDER Statement Checklist

| **Item** | **Checklist Item** | **N studies (%)** | **Supporting Study**  **(Reference)** |
| --- | --- | --- | --- |
| **Domain 1. Governance** | | | |
| 1 | a: Describe partnership agreements between the research institution & Indigenous-governing organisation/s | 1 (14.29%) | Campbell 2013 |
| 2 | b: Describe accountability and review mechanisms within the partnership agreement that addresses harm minimisation | 0 |  |
| 3 | c: Specify how the research partnership agreement includes protection of Indigenous intellectual property and knowledge arising from the research, including financial and intellectual benefits generated | 0 |  |
| **Domain 2. Prioritization** | | | |
| 4 | a: Explain how the research aims emerged from priorities identified by either Indigenous stakeholders, governing bodies, funders, non-government organization(s), stakeholders, consumers, and empirical evidence | 7 (100%) | Best 2009, Best 2012, Brennand 2005, Campbell 2013, Daly 2018, Sina 2014, Thrift 2014 |
| **Domain 3. Relationships (Indigenous stakeholders/participants and Research team)** | | | |
| 5 | Specify measures that adhere to and honour Indigenous ethical guidelines, processes & approvals for all relevant Indigenous stakeholders, recognizing that multiple Indigenous partners may be involved, e.g., Indigenous ethics | 1 (14.29%) | Daly 2018 |
| 6 | Report how Indigenous stakeholders were involved in the research processes (i.e., research design, funding, implementation, analysis, dissemination/recruitment). | 0 |  |
| 7 | Describe the expertise in Indigenous health and research of the research team. | 1 (14.29%) | Campbell 2013 |
| **Domain 4. Methodologies** | | | |
| 8 | Describe the methodological approach of the research, including a rationale of methods used and implication for Indigenous stakeholders eg privacy and confidentiality (individual and collective) | 0 |  |
| 9 | Describe how the research methodology incorporated consideration of the physical, social, economic, and cultural environment of the participants and prospective participants. (e.g., impacts of colonization, racism, and social justice). As well as Indigenous worldviews. | 0 |  |
| **Domain 5. Participation** | | | |
| 10 | Specify how individual and collective consent was sought to conduct future analysis on collected samples and data, other than what was the approved initially (e.g., third parties accessing samples (genetic, tissue, blood) for additional analyses). | 4 (57.14%) | Best 2009, Best 2012, Campbell 2013, Daly 2018 |
| 11 | Provide details on how the resource demands (current and future) placed on Indigenous participants and communities involved in the research were identified and agreed upon including any resourcing for participation, knowledge and expertise | 0 |  |
| 12 | Specify how biological tissue and other samples, including data, were stored/disposed of. | 0 |  |
| **Domain 6. Capacity** | | | |
| 13 | Explain how the research supported the development and maintenance of Indigenous research capacity | 0 |  |
| 14 | Discuss how the research team undertook professional development opportunities to develop the capacity to partner with Indigenous stakeholders | 0 |  |
| **Domain 7. Analysis and Interpretation** | | | |
| 15 | Specify how the research analysis and reporting supported critical inquiry and a strength-based approach that was inclusive of Indigenous values | 0 |  |
| **Domain 8. Dissemination** | | | |
| 16 | Describe how the research findings were disseminated to relevant Indigenous governing bodies and peoples. | 1 (14.29%) | Brennand 2005 |
| 17 | Discuss the process for knowledge translation and implementation to support Indigenous advancement | 1 (14.29%) | Campbell 2013 |

# SUPPLEMENTARY Material 3, Table 2: Studies Reporting Items in the CREATE Statement Checklist

| **Item** | **Checklist Item** | **N studies (%)** | **Supporting Study**  **(Reference)** |
| --- | --- | --- | --- |
| 1 | Q1: Did the research respond to a need or priority determined by the community? | 0 |  |
| 2 | Q2: Was community consultation and engagement appropriately inclusive? | 0 |  |
| 3 | Q3: Did the research have Aboriginal and Torres Strait Islander [or Indigenous] research leadership? | 1 (14.29%) | Daly 2018 |
| 4 | Q4: Did the research have Aboriginal and Torres Strait Islander [or Indigenous] governance? | 0 |  |
| 5 | Q5: Were local community protocols respected and followed? | 1 (14.29%) | Campbell 2013 |
| 6 | Q6: Did the researchers negotiate agreements in regards to rights of access to Aboriginal and Torres Strait Islander [or Indigenous] peoples’ existing intellectual and cultural property? | 0 |  |
| 7 | Q7: Did the researchers negotiate agreements to protect Aboriginal and Torres Strait Islander [or Indigenous] peoples’ ownership of intellectual and cultural property created through the research? | 0 |  |
| 8 | Q8: Did Aboriginal and Torres Strait Islander [or Indigenous] peoples and communities have control over the collection and management of research materials? | 0 |  |
| 9 | Q9 Was the research guided by an Indigenous research paradigm? | 0 |  |
| 10 | Q10: Does the research take a strengths-based approach, acknowledging and moving beyond practices that have harmed Aboriginal and Torres Strait peoples in the past? | 0 |  |
| 11 | Q11: Did the researchers plan and translate the findings into sustainable changes in policy and/or practice? | 2 (28.57%) | Brennand 2005, Campbell 2013 |
| 12 | Q12: Did the research benefit the participants and Aboriginal and Torres Strait Islander [or Indigenous] communities? | 2 (28.57%) | Brennand 2005, Campbell 2013 |
| 13 | Q13: Did the research demonstrate capacity strengthening for Aboriginal and Torres Strait Islander [or Indigenous] individuals? | 0 |  |
| 14 | Q14: Did everyone involved in the research have opportunities to learn from each other? | 0 |  |

**Note**: The Authors applied this tool – designed for use within research involving Aboriginal and Torres Strait Islander peoples – to all research papers identified in our searching. As such, where the tool asks if Aboriginal and Torres Strait Islander peoples’ were considered, for papers from outside Australia the authors considered whether the geographically relevant Indigenous Peoples were considered.
